# 2D Ti_3_C_2_T_x_ (MXene)-reinforced polyvinyl alcohol (PVA) nanofibers with enhanced mechanical and electrical properties

**DOI:** 10.1371/journal.pone.0183705

**Published:** 2017-08-30

**Authors:** Patrik Sobolčiak, Adnan Ali, Mohammad K. Hassan, Mohamed I. Helal, Aisha Tanvir, Anton Popelka, Mariam A. Al-Maadeed, Igor Krupa, Khaled A. Mahmoud

**Affiliations:** 1 Center for Advanced Materials, Qatar University, Doha, Qatar; 2 Qatar Environment and Energy Research Institute (QEERI), Hamad Bin Khalifa University (HBKU), Doha, Qatar; 3 Materials Science and Technology Program, Qatar University, Doha, Qatar; 4 QAPCO Polymer Chair, Center for Advanced Materials, Qatar University, Doha, Qatar; 5 Department of Physics & Mathematical Engineering, Faculty of Engineering, Port Said University, Port Said, Egypt; Institute of Materials Science, GERMANY

## Abstract

Novel 2D Ti_3_C_2_T_x_ (MXene)-reinforced polyvinyl alcohol (PVA) nanofibers have been successfully fabricated by an electrospinning technique. The high aspect ratio, hydrophilic surfaces, and metallic conductivity of delaminated MXene nanosheet render it promising nanofiller for high performance nanocomposites. Cellulose nanocrystals (CNC) were used to improve the mechanical properties of the nanofibers. The obtained electrospun nanofibers had diameter from 174 to 194 nm depending on ratio between PVA, CNC and MXene. Dynamic mechanical analysis demonstrated an increase in the elastic modulus from 392 MPa for neat PVA fibers to 855 MPa for fibers containing CNC and MXene at 25°C. Moreover, PVA nanofibers containing 0.14 wt. % Ti_3_C_2_T_x_ exhibited dc conductivity of 0.8 mS/cm conductivity which is superior compared to similar composites prepared using methods other than electrospinning. Improved mechanical and electrical characteristics of the Ti_3_C_2_T_x_ /CNC/PVA composites make them viable materials for high performance energy applications.

## 1. Introduction

The demand for highly conductive, lightweight and flexible electrodes for various energy applications has sparked interest in developing new methods to fabricate freestanding and flexible films, containing minimum inactive ingredients. Nanofibers prepared by electrospinning (ES) are of considerable interest for various applications due to their unique nanofibrous structure, large surface area, and high porosity [1–3].

Electrospun polyvinyl alcohol (PVA) fibers are of particular interest due to their biocompatibility and biodegradability [4, 5]. PVA has good transparency and anti-electrostatic properties [6]. The hydrophilic nature of PVA-based films is useful for applications like desalination membranes due to reduced fouling by nonpolar molecules, microbes and fulvic acids [7]. However, the barrier and thermal properties as well as mechanical strength of (cross-linked) fibers are poor. In order to improve those properties, hydrophilic fillers such as iron oxide [8], sodium montmorillonite clay particles [9], and cellulose nanocrystals (CNCs) [10] are incorporated in the continuous polymeric matrix. CNCs are particularly interesting because of their environmental friendliness, high mechanical performance, flexibility, low-cost, versatility, and tailorable surface functionalities [11–13]. The size, structure, and functional groups of CNC are dependent on the source of cellulosic fibres and preparation method [14–17].

1D and 2D carbon nanomaterials based nano fillers including graphite nanoplatelets, graphene oxide (GO), carbon nanotubes (CNT), and graphene [18–20] have been used to reinforce electrospun fibers and enhance their electrical and mechanical properties for many applications, including mechanically reinforced composites [21], fuel cell applications [22], separation membranes [23], supercapacitors [24], lithium-ion batteries [25] and photocatalytic degradation [26].

Very recently, a new class of 2D metal carbides and carbonitrides called MXenes, which are both conductive as well as hydrophilic have been discovered [27]. MXenes have general formula M_n+1_X_n_, which is derived from MAX phases, where M is an early transition metal, A is an A-group element, mostly IIIA and IVA, or groups 13 and 14, and X is either carbon and/or nitrogen, by chemical etching in HF or NH_4_HF_2_ solutions, where n = 1, 2 or 3 [28], The unique structure of MXenes offers combination of excellent mechanical properties, hydrophilic surface, transparency and metallic conductivity [29].

Recently, MXenes have been used as promising electrodes, since they can spontaneously adsorb proper cations with potential redox reactions on active sites, thereby permitting relatively large capacity of corresponding batteries [30].

MXenes can not only store large amounts of electrical charges, but also discharge at rapid rates, which is in sharp controversy to other conventional anodes materials.

Ti_3_C_2_T_x_ is well-studied MXene, especially in supercapacitors. In an early attempt, flexible Ti_3_C_2_T_x_ paper was prepared as the electrodes with a volumetric capacitance of 350 F cm^-3^ in NaOH solution [31].

Ghidiu et al. reported flexible and free-standing films from clay-like MXene paste [32].

The resultant flakes posse larger lateral dimensions with high volumetric capacitance of 900 F×cm^-3^ and almost 100% retention even after 10,000 cycles [32].

Recently, Ti_3_C_2_T_x_/polymer composite film with high volumetric capacitance was produced by simple of mixing Ti_3_C_2_T_x_ and PVA [33]. In comparison to neat PVA or Ti_3_C_2_T_x_ films, the flexible Ti_3_C_2_T_x_/PVA composite film showed an increase of mechanical strength, a high conductivity and an outstanding volumetric capacitance as high as 528 F×cm^-3^ at 2 mV×s^-1^. Sandwich-like MXene/CNT composite paper electrodes were fabricated in layer-by-layer form through alternate filtration of MXene and CNT dispersions and results showed a volumetric capacitance of 350 F×cm^-3^ at 5 A×g^-1^ without degradation even after 10000 cycles [34].

Herein, we report for the first time the fabrication of electrospun fibers based on Ti_3_C_2_T_X_/PVA and Ti_3_C_2_T_X_/CNC/PVA composite. A benefit of using two different fillers is an ability to tailor properties (such as mechanical, thermal, electrical etc.) of PVA fibres regarding their specific target application. The electrospun nanofibers were analyzed using scanning electron microscopy (SEM), atomic force microscopy (AFM), dynamic mechanical analysis (DMA), tensile measurement, thermogravimetric measurement (TGA) and broadband dielectric spectrometry (BDS).

## 2. Materials and methods

### 2.1 Materials

PVA (Sigma-Aldrich, Mw around 90 kDa), NaOH (Sigma Aldrich), (NH_4_)_2_S_2_O_8_ (Sigma Aldrich), ethanol (Sigma-Aldrich), date palm leaves (*Phoenix dactylifera*), DI water was used as a solvent. Multilayer ML-Ti_3_C_2_T_x_ was obtained from Nanomaterials Institute, Drexel University.

### 2.2 Preparation of carboxylated nanocellulose (CNC)

CNC was prepared from date palm leaves (*Phoenix dactylifera*) by slightly modified previously reported method [17]. Briefly, date palm leaves (10 g) were grinded to a fine powder and added to 1 L of 1 M ammonium persulfate solution. The mixture was heated to 60°C and was vigorously stirred overnight to yield a white suspension of CNCs. The suspension was centrifuged (5000 rpm for 10 min. and washed with DI water). The centrifugation/washing cycles were repeated until the solution pH close to 4. The product was lyophilized to yield a white powder. CNCs in their sodium form were prepared by slow titration with 1 M NaOH until the suspension reaches pH ≈ 7, followed by washing/centrifugation with deionized water. Purity and crystallinity were confirmed by TEM and XRD (S1A and S2A Figs, S1 File).

### 2.3 Preparation of delaminated Ti_3_C_2_T_x_ sheets

Multi-layer (ML-Ti_3_C_2_T_x_) powder was dispersed in deaerated 70% ethanol/water with a weight ratio of ML-Ti_3_C_2_T_x_: ethanol 250:1. The suspension was sonicated (20 minutes at 60% amplitude while 3 sec pulse on and 1 sec off) under flow in argon, and then centrifuged at 3000 rpm for 1 h to obtain the supernatant containing Ti_3_C_2_T_x_ flakes (1.16 mg/ml). The electrical conductivity of the Ti_3_C_2_T_x_ ink 19.4 μS×cm^-1^ was measured using a conductivity meter (Cond6+ meter). Purity and crystallinity were confirmed by TEM and XRD (S1 File, S1B and S2B Figs).

### 2.4 Electrospinning of composite fibers

PVA was dissolved in distilled water at 80°C for at least 2 hours to obtain 15 wt.% solution. Ti_3_C_2_T_X_ and CNC were added to PVA solution followed by dispersion by ultra-sonication for 20 min in an ice bath. Compositions of prepared solutions, together with viscosity (measured at room temperature by vibro viscometer SV10) and conductivity (measured at room temperature by Thermo Scientific Orion 013005MD Conductivity Cell) are summarized in Table 1. All suspensions were visually inspected to confirm good dispersion of Ti_3_C_2_T_X_ and CNC particles.

**Table 1:** Composition of prepared 15 wt.% PVA solutions.

Sample		* CNC (wt.%)	* Ti_3_C_2_T_X_ (wt.%)	Viscosity (Pa×S)	Conductivity (μS/cm)
C_0_M_0_	CNC_0_/ Ti_3_C_2_T_X 0_	0	0	2.33	538
C_2_M_0_	CNC_0.02_/ Ti_3_C_2_T_X 0_	0.14	0	2.64	545
C_0_M_2_	CNC_0_/ Ti_3_C_2_T_X 0.02_	0	0.14	2.47	590
C_1_M_1_	CNC_0.01_/ Ti_3_C_2_T_X 0.01_	0.07	0.07	2.63	564

* Concentration of fillers related to concentration of PVA solution

Electrospun nanofibers were fabricated using NaBond electrospinning (Shenzhen, China). Typically, 5 mL PVA suspensions were loaded into a 10-mL syringe, with a blunt-end, stainless steel needle. An aluminium foil covered rotating drum collector was used as the collection screen, it was connected to the ground electrode of the power supply with 10 cm distance between the screen and the needle tip. The electrospinning process was carried out at room temperature at the voltage 17 kV, flow rate 0.3 mL/h and drum speed 200 RPM. The thickness of prepared mats was around 50 μm. Subsequently, electrospun mats were visually checked to confirm no defect or drops of the PVA solution that may potentially affect further characterization.

### 2.5 Scanning electron microscopy (SEM)

The surface morphology of the Ti_3_C_2_T_X_/CNC/PVA fibers was examined by a field emission scanning electron microscope (FE-SEM, Nova Nano SEM 650) equipped with Energy-dispersive X-ray spectroscopy (EDS) by Secondary electron images with 3 kV and different magnifications. All specimens were sputter-coated with 2 nm gold before the use of the SEM. The average thickness of the fibers was measured by image J software.

### 2.6 Atomic force microscopy (AFM)

AFM was used to further elucidate 2D and 3D surface morphology of electrospun nanofibers using MFP3D Asylum research (USA) equipped with a Silicon probe (Al reflex coated Veeco model–OLTESPA, Olympus; spring constant: 2 N×m^-1^, resonant frequency: 70 kHz). Measurements were performed under ambient conditions using the Standard Topography AC air (tapping mode in air). An AFM head scanner applied with Si cantilever adjacent vertically in the sample resonant frequency of the free-oscillating cantilever set as the driving frequency.

### 2.7 Dynamic mechanical analysis (DMA)

Effect of Ti_3_C_2_T_X_ and CNC loadings on the viscoelastic properties of PVA was studied using DMA (RSA-G2, TA Instruments, USA) in tensile mode at 25°C and ambient atmosphere. Rectangular samples 15 mm×3 mm×0.05 mm were cut from the prepared samples. The linear viscoelastic range was determined from the constant value of the elastic modulus on 0.1–200 μm displacement and 30–100°C temperature sweep at a heating rate of 5°C min^-1^ and at 1 Hz frequency. The specimens were subjected to a tensile deformation of 0.1%.

### 2.8 Tensile measurement

Nanofibers mats were cut into narrow strips and twisted into short nanofiber yarns using the Cord maker & Fringe twister II (LACIS, CA, USA). In order to ensure uniform thickness, 5cm width strips were twisted for 20 seconds each. The twisted fibers were mounted on a C-shaped mechanical testing card with a 50 mm distance between C shape shoulders (See S1 File for more detail of sample preparation). The diameter of the twisted fibers yarns was measured using a SEM and nine readings, along the length of the yarn, were averaged to calculate the diameter of the yarn. Mechanical testing was done at room temperature using the LF LLOYD INSTRUMENTS (An Ameter Company) using a 10 g load cell (Transducer Techniques; Temecula, CA) with a pulling rate of 10 mm/min. Each sample was measured at least 10 times and average values with standard deviations were calculated to analyse mechanical properties of fibers.

### 2.9 Thermogravimetric measurement (TGA)

TGA measurements were performed using TGA 4000 (Perkin Elmer, USA) at temperature range from 30°C to 600°C at a heating rate of 20°C/min under nitrogen atmosphere. Nitrogen gas was passed through the instrument at a flow rate of 20 ml×min^−1^. The weight of the samples varied from 10 to 15 mg.

### 2.10 Broadband dielectric spectroscopy (BDS)

Dielectric measurements were performed using a Novocontrol GmbH Concept 40 broadband dielectric spectrometer (Montabaur, Germany), and data were collected over the frequency range 0.1Hz–3MHz at fixed temperatures in the range of -70 to 200°C. Sample discs of 2 cm diameter were sandwiched between two gold-coated copper electrodes of 2 cm diameter and then transferred to the instrument for data collection. The AC conductivity was calculated, from the Novocontrol WinDeta software, by using the measured values of dielectric permittivity and the dielectric loss factor.

## 3. Results and discussion

### 3.1 Morphological characterization

A mixed solution of PVA containing different wt % of Ti_3_C_2_T_x_ and CNC was used to fabricate the electrospun nanofibers. Ti_3_C_2_T_x_CNC/PVA aqueous blends possess good physicochemical properties (Table 1). As expected, viscosity and conductivity of the blends have increased with higher Ti_3_C_2_T_x_ and CNC amounts, respectively. The well-dispersed aqueous solutions were directly electrospun to form the mixed ratio nanofibers described in Table 1.

**Fig 1:**
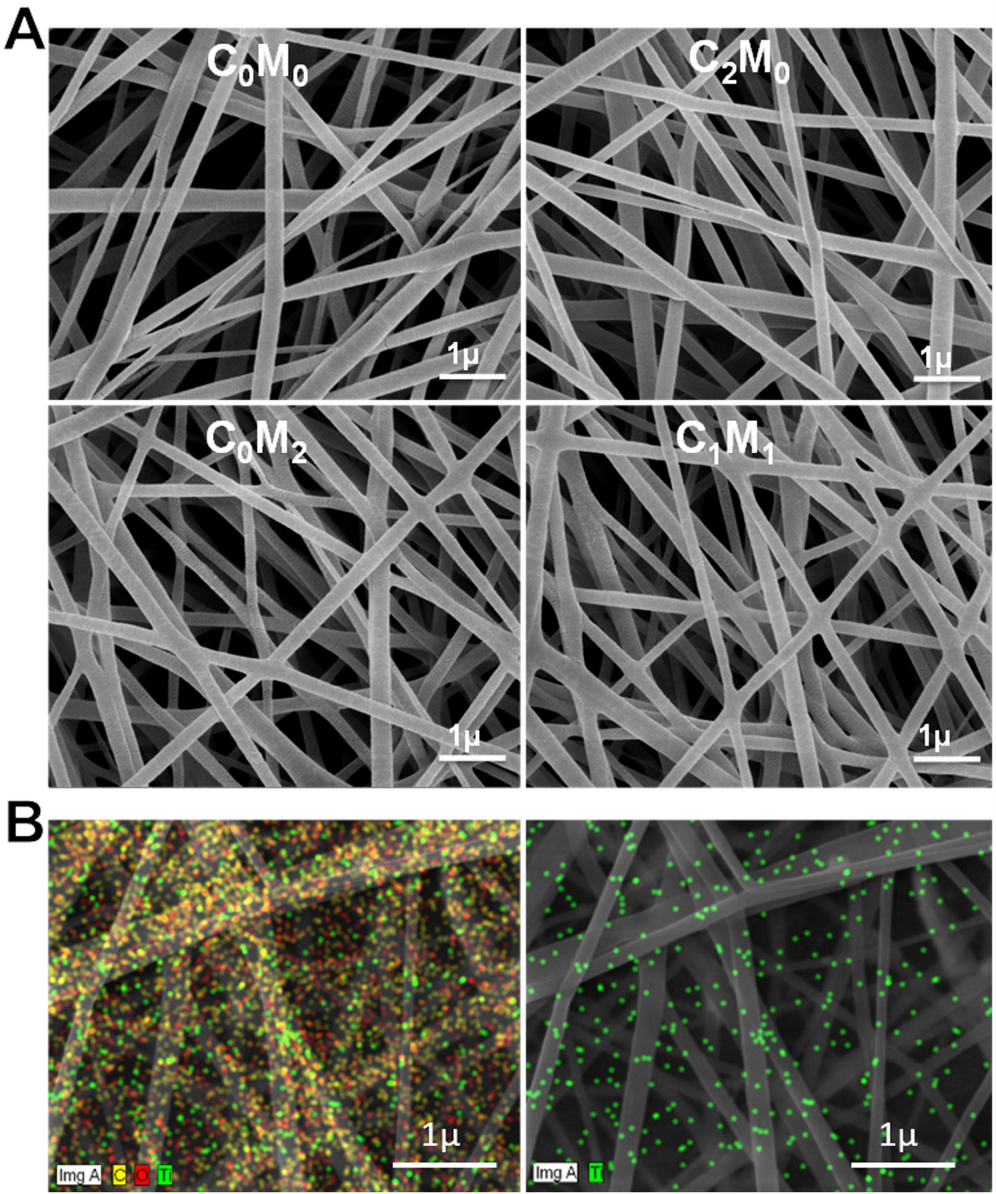
SEM images of electrospun nanofibers. A) SEM images of the reinforced PVA nanofibers at different loading of CNC and Ti_3_C_2_T_X_ (Table 1), B) dispersion pattern of Ti_3_C_2_T_X_ shown by EDS mapping of sample C_0_M_2_ where; C (yellow), O (oxygen) and Ti (green).

The representative SEM micrographs of the four nanofibers composites are shown in Fig 1a and described in Table 1.

The obtained nanofibers appear to be uniform and smooth with thickness between 174 to 194 nm with no beads and uniform distribution of fibres thickness. As the ratio of Ti_3_C_2_T_X_ increases in the composite the nanofibers diameter decreased as clearly seen for blends C_1_M_1_, C_0_M_2_ as compared with those containing only PVA (C_0_M_0_). The average diameters of the nanofibers measured from SEM were 182 (±53), 195 (±33), 174 (±53), 179 (±36) nm for C_0_M_0_, C_2_M_0_, C_0_M_2_ and C_1_M_1_ respectively. The presence of Ti_3_C_2_T_x_ within C_0_M_2_ and C_1_M_1_ electrospun nanofibers was confirmed by tracing Ti element in the EDS spectra. (S3 Fig; S1 File).

Fig 1b shows EDS mapping of C_0_M_2_ containing Ti_3_C_2_T_X_ (green color) where good dispersion of Ti_3_C_2_T_X_ particles within PVA matrix were confirmed.

**Fig 2:**
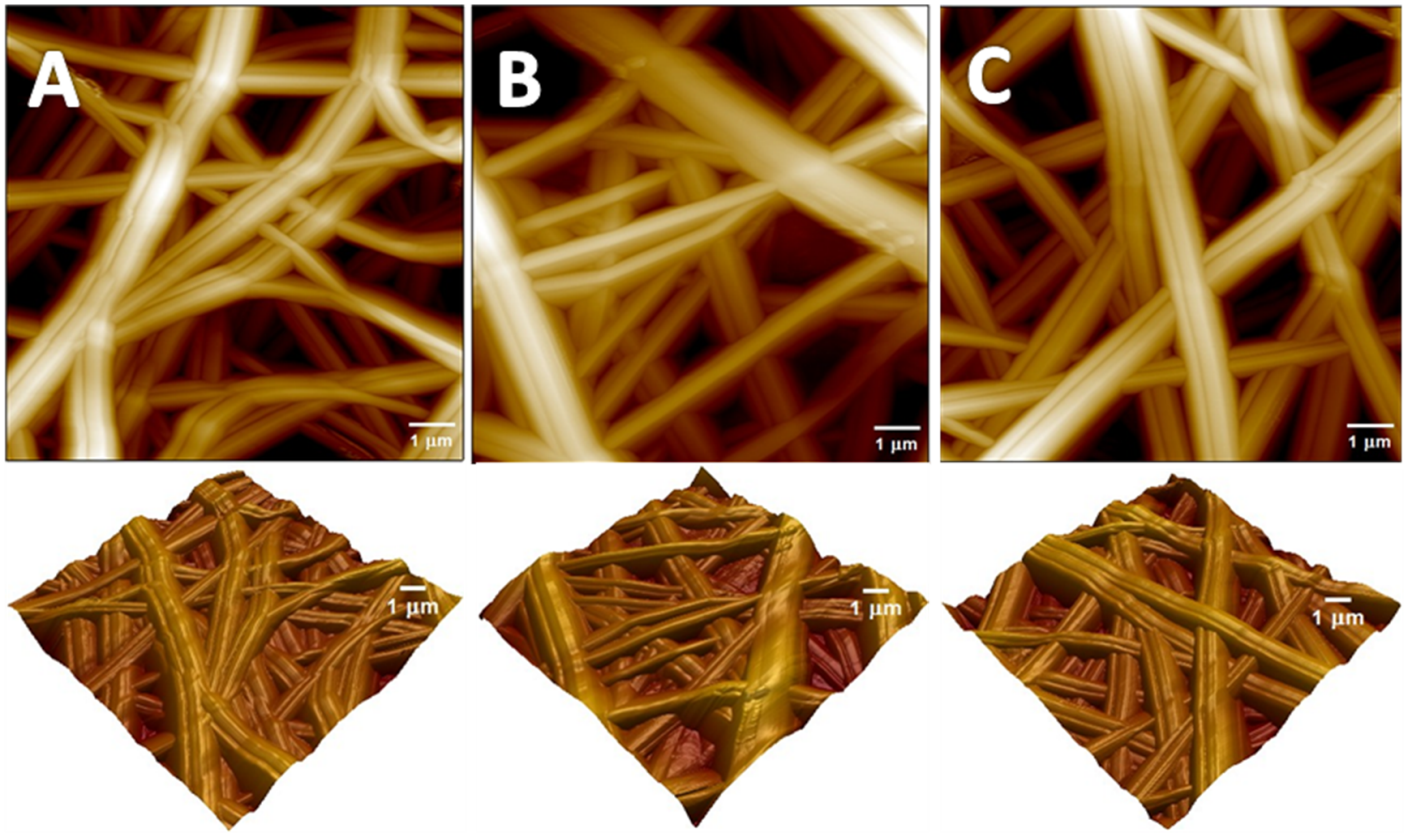
2D and 3D AFM phase images of A) C_2_M_0_, B) C_0_M_2_ and C) C_1_M_1_.

AFM was used to further elucidate the surface morphology of the fibers as described in Fig 2. The 2D AFM-phase and 3D AFM-height images were obtained using intermittent-contact (tapping) mode. The C_2_M_0_, C_0_M_2_ and C_1_M_1_ samples exceled axially oriented structures of fibers with relatively smooth surfaces. No visible aggregation was observed in the presence of CNC or Ti_3_C_2_T_X_ which were sufficiently located inside the fibers. Moreover, no significant differences of roughness have been found in longitudinal as well as lateral directions ensuring the well dispersion of them within the PVA fibers. Some of the fibers were bundled together leading to the formation of multi-fiber structures. In all cases, the prepared fibers led to the formation of interlaced structures responsible for the creation of relative regular networks.

### 3.2 Mechanical characterization

Mechanical characterization of electrospun nanofibers was performed by dynamic mechanical measurement and tensile measurement.

**Fig 3:**
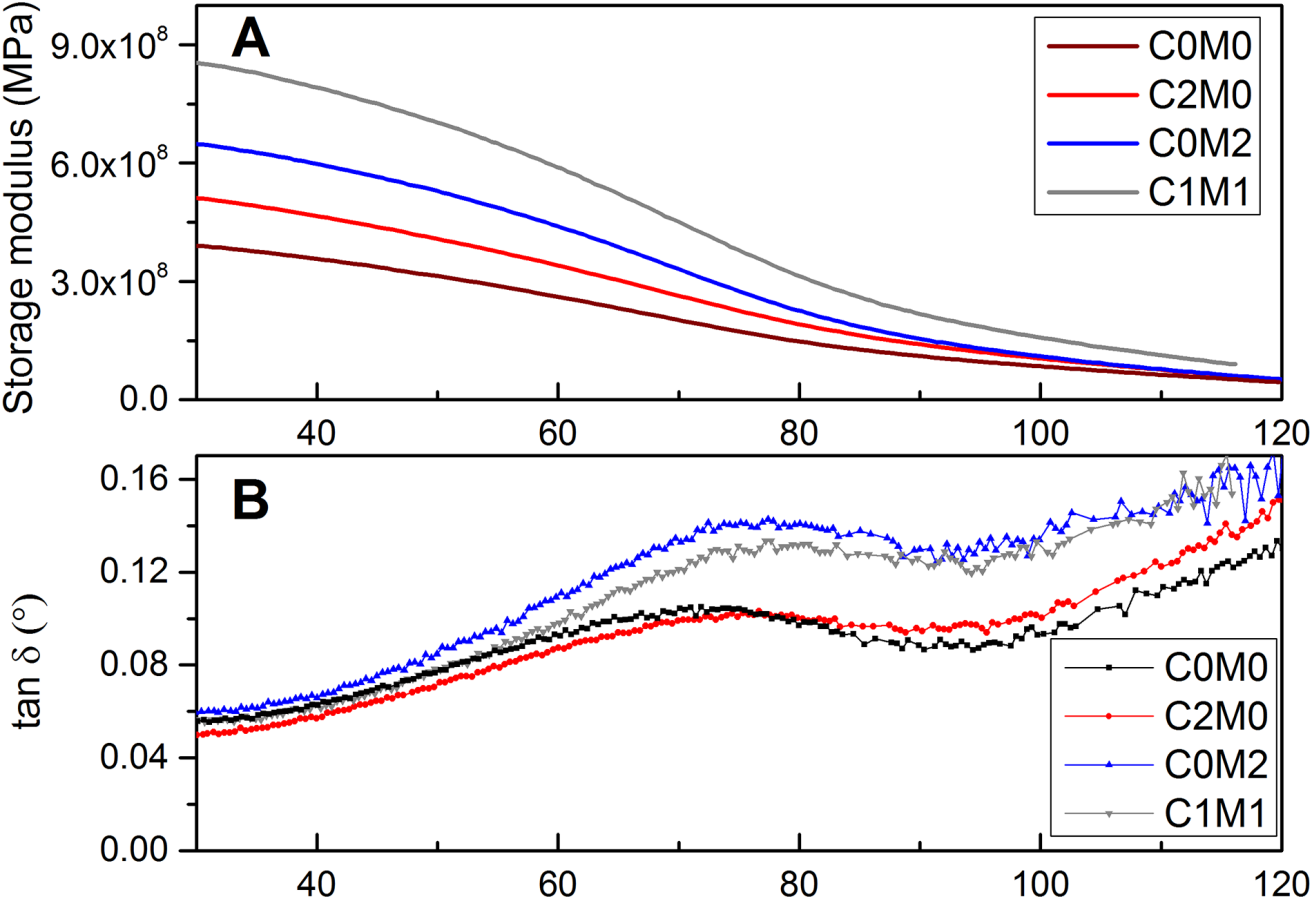
Dynamic mechanical analysi of electrospun mats. A) Storage modulus of PVA samples contain CNC and Ti_3_C_2_T_X_ fillers; B) tan δ of PVA fibres contain CNC and Ti_3_C_2_T_X_ fillers.

The dynamic mechanical behaviour of the electrospun mats containing CNC or/and Ti_3_C_2_T_X_ fillers was measured in the broad temperature range (Fig 3A) to provide a complex investigation of the prepared electrospun nanofibers. Storage modulus increases in order: neat PVA, CNC/PVA and Ti_3_C_2_T_X_/PVA what indicated the reinforcement effect of CNC and Ti_3_C_2_T_X_ within PVA fibers. The comparison of storage modulus of samples containing 0.14 wt % either CNC or Ti_3_C_2_T_X_ with sample containing both fillers with concentration 0.07 wt. % each indicated synergic effect of CNC and Ti_3_C_2_T_X_ fillers.

Indeed, the nanofibers containing 0.14 wt % CNC exhibited around 20% increasing of storage modulus compare to neat PVA fibers. In addition, nanofibers containing 0.07 wt.% of both, CNC and Ti_3_C_2_T_X_ fillers exhibited over 100% increase in of storage modulus compare to PVA nanofibers at 25°C, what is superior compared to previously reported study [35], where 74% increase in storage modulus of PVA film by addition of 3 wt.% nanocellulose at 25°C have been reported.

Our hypothesis is that the self-orientation of CNC nanofibers during electrospinning within PVA matrix caused by the electrical field has remarkable influence on the mechanical properties of the nanofibers composites.

Furthermore, Fig 3B is the tan δ curve, which is the ratio of loss modulus to storage modulus and is a measure of the damping behaviour of a material [11]. Fig 3Ba showed a shift in the glass transition temperature with addition of both CNC and Ti_3_C_2_T_X_ fillers.

Glass transition temperature (*T*_g_) of neat PVA nanofibers was 70.2°C. The addition of CNC resulted in an increase of the *T*_g_ to 79.1°C indicating significant improvement of mechanical response for this type of polymer. The addition of Ti_3_C_2_T_X_ to PVA matrix led to increasing of *T*_g_ up to 80.5°C. Furthermore, PVA nanofibers containing CNC and Ti_3_C_2_T_X_ (C_1_M_1_) assigned *T*_g_ 82.3°C which also support hypothesis about synergic effect imparted by both fillers.

**Fig 4:**
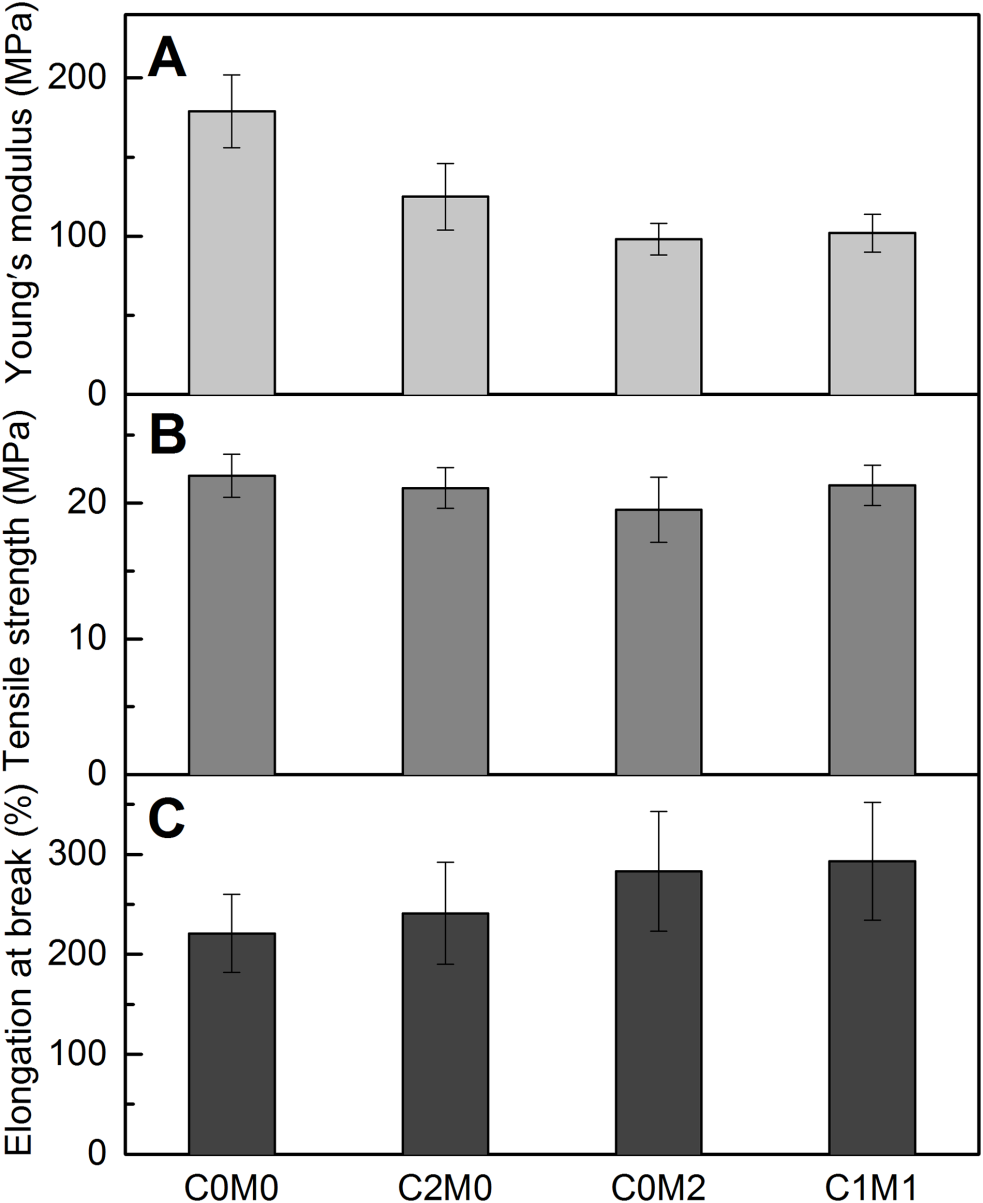
Mechanical properties of prepared PVA electrospun nanofibers. Young´s modulus, B) Tensile strength and C) Elongation at break.

As a second approach to study mechanical properties of prepared nanofibers, tensile test has been performed. Obtained Young’s modulus, tensile strength and elongation at break for PVA electrospun composites are listed in Fig 4. A slight increase of Young’s modulus of PVA electrospun mats with addition CNC fillers, from 221±51 MPa to 241±51 MPa was observed. Sample containing Ti_3_C_2_T_X_ particles showed additional increase of Young’s modulus 283±60 MPa and sample containing both fillers showed Young’s modulus of 293±59 MPa. Young’s modulus obtained from tensile measurement is in line with storage modulus obtained from DMA study. However, absolute values cannot be compared due to nature of the experiment and sample preparation. Tensile measurements were performed for twisted samples to eliminate effect of mostly randomly orientated nanofibers within electrospun mat, whereas DMA measurement were done for specimens cutted from electrospun mats without twisting.

On the other hand, only slight dependence of tensile strength on the filler content 19.5±2.4 to 22.01.3±1.6 MPa was observed.

The elongation at break decreased with addition of the fillers, a common behaviour for polymeric composites. Elongation at break for neat PVA was 179 ±23% whereas elongation for samples containing fillers ranging from 98±10% to 125 ±21%.

It can be concluded that addition of CNC improves mechanical performance of composites probably due to its highly crystalline structure and high aspect ratio [35], whereas Ti_3_C_2_T_X_ also tend to have high mechanical performance and contributes to increase in the of mechanical properties of the nanofibers [33].

### 3.3 Thermogravimetric measurement

Thermal gravimetric analysis (TGA) is commonly used technique to investigate thermal decomposition behaviours of polymers at different temperatures, and also demonstrates thermal-resistant of various materials.

**Fig 5:**
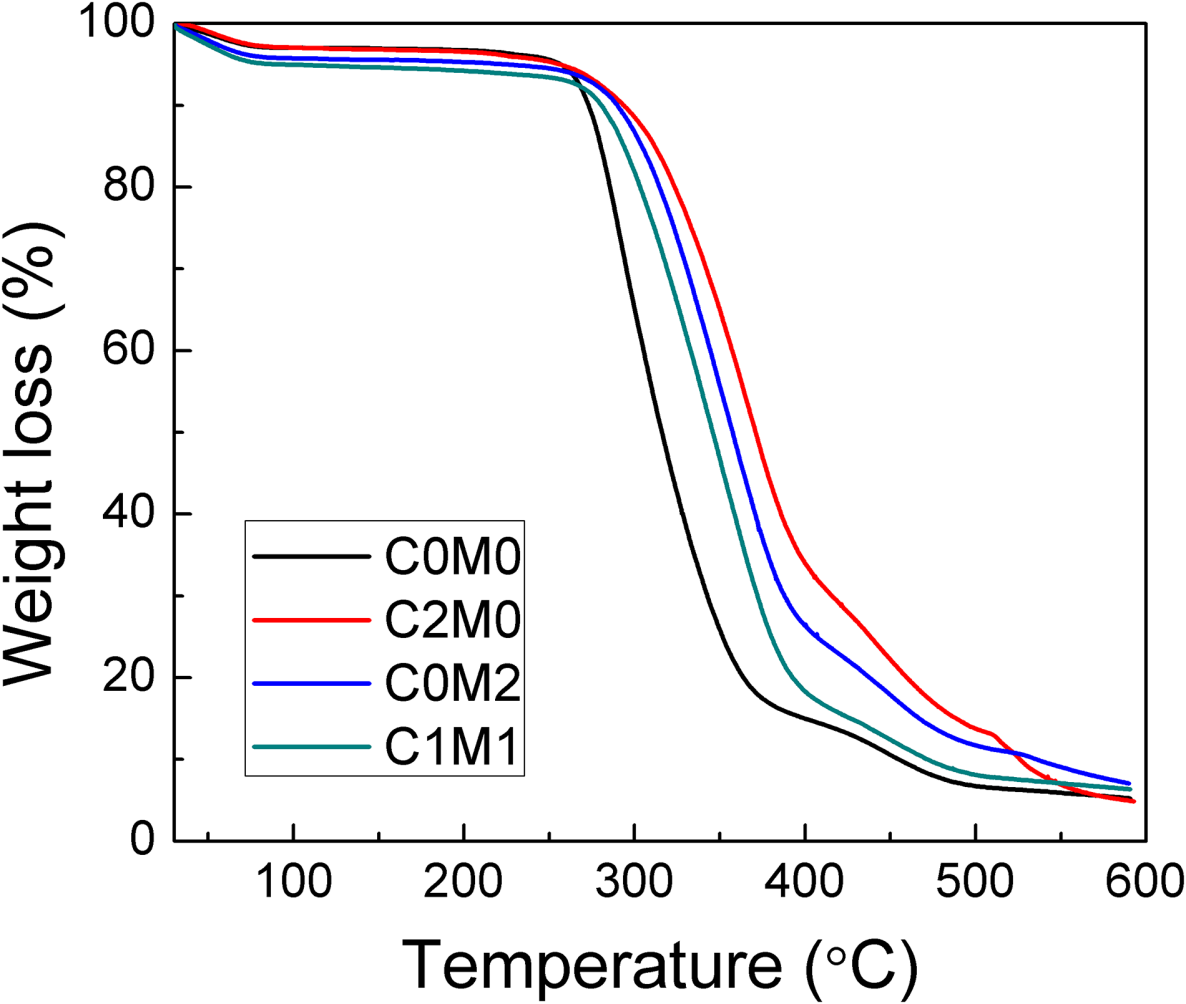
TGA of PVA electrospun nanofibers containing CNC and Ti_3_C_2_T_X_ fillers.

TGA measurement of all prepared electrospun composites is shown in Fig 5. First weight loss, at the temperature up to 100°C, occurred due to evaporation of moisture since PVA is known as a hydrophilic polymer. The most expressive weight-loss (more than 70%) occurs from 245°C to 380°C, due the breakdown of the polymer The third weight-loss step, of about 20%, is observed between 380°C to 470°C, which can be associated mainly with the PVA degradation. The onset temperature of PVA degradation is 270.2°C with peak maximum at the temperature 287°C. The addition of CNC caused the most significant enhancement of thermal properties of PVA, where the onset temperature shifts 343.5°C and peak maximum to 367.7°C. Similar results, increasing of thermal properties of PVA by adding nanocellulose were reported previously [36, 37].

MXene particles also caused increasing of thermal stability of the electrospun PVA nanofibers compared to neat PVA with onset temperature 290.9°C and peak maximum at the temperature 354.9°C. In addition, PVA containing both fillers, MXene and CNC assigned onset temperature 282.5 and maximum peak degradation at 345.6°C.

Herein, it can be stated that MXene as well as CNC caused remarkable enhancement of thermal properties of electrospun PVA nanofibers.

### 3.4 Conductivity characterization

**Fig 6:**
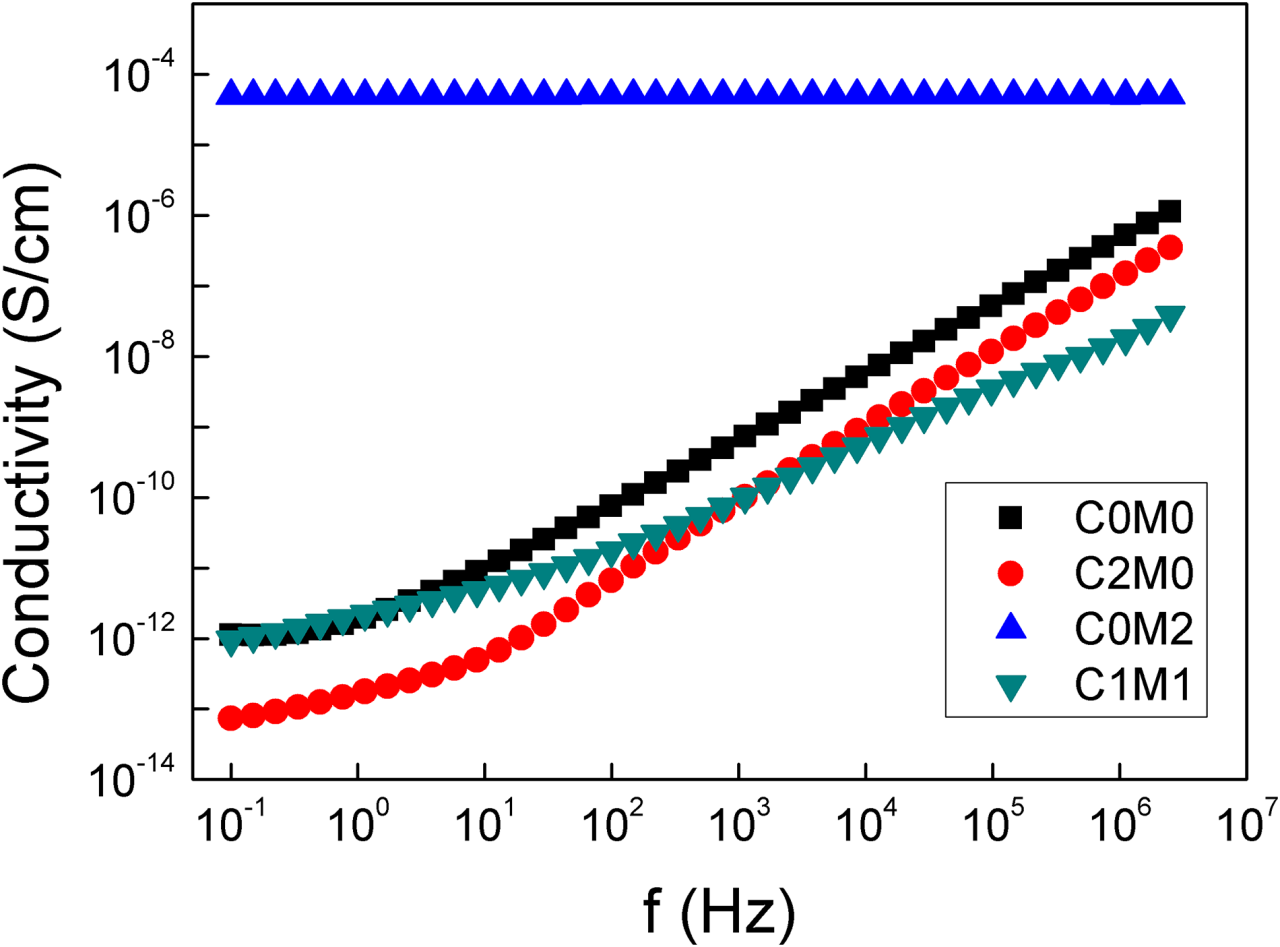
DC conductivity vs. frequency at 20°C for the Ti_3_C_2_T_x_/CNC/PVA nanofiber samples with different composition.

The dc conductivity (σ′) of the Ti_3_C_2_T_x_/CNC/PVA nanofiber composites is given in Fig 6. DC conductivity exhibited the typical behaviour for polymers and disordered solids as it has frequency independence at low frequencies then rises monotonically with the s-th power of frequency, where s ranges between 0.7 and 1.0 [13, 38, 39].

The conductivity shows a plateau at low frequency which corresponds to the dc conductivity and the frequency at which it becomes frequency dependent is called the relaxation frequency, ω_s_ [40].

The dc conductivity increases and the plateau widen to include the entire frequency range for high MXene concentration sample C_0_M_2_. The results revealed a highly conductive composite film with excellent mechanical characteristics. In addition to being conductive filler, Ti_3_C_2_T_x_ acted as a reinforcing component, along with the CNC, causing enhancements in the mechanical properties of the PVA fibers. Similarly, Zhang et al. used Ti_3_Si_0.75_Al_0.25_C_2_ and Ti_3_Al_0.9_Si_0.1_C_2_ nanosheets to prepare poly(methyl methacrylate) (PMMA) based composites with great enhancements in the mechanical and thermal properties [41]. Their composites showed fivefold increase in the Young modulus and twofold increase in the tensile strength comparing to the control PMMA. They revealed that the obtained mechanical characteristics enhancements were much higher than those obtained with the boron nitride/methyl methacrylate and the graphene/PMMA composites having the same weight percent of nanosheets [41]. They also revealed that the nanosheets improved the thermal conductivity of the composites but no reference to their electrical conductivity.

In PVA, electron conduction occurred by hopping through the film due to polymer polarization to transport electrons [42]. The presence of CNC would cause large resistivity to these hopping and thus result in conductivity reduction observed in Fig 5 for sample C_2_M_0_. It is also worth noting that the conduction behaviour exhibited by sample C_1_M_1_ is almost the same as that of the control PVA although it contains the conductive MXene nanosheets. However, small amount of Ti_3_C_2_T_x_ does not seem to be enough to achieve the connected conductive pathway or it is below the percolation threshold for the conductivity in these composites.

Ti_3_C_2_T_x_ was used recently by Ling et al. to prepare flexible/mechanically strong PVA composites films by simple mixing and solvent casting technique. They were investigated as electrodes for supercapacitors and demonstrated a notable volumetric capacitance in KOH electrolyte [30]. Although these Ti_3_C_2_T_x_/PVA films showed excellent conductivity; the authors used very high amounts of Ti_3_C_2_T_x_ in preparing the composites [30]. For example, a sample containing 40 wt% of Ti_3_C_2_T_x_ revealed a conductivity of 40 mS/cm. This amount of MXene is much higher comparing to the amounts used in the work presented herein. Sample C_0_M_2_ in our study exhibits a conductivity of ≅ 0.08 mS/cm, with only 0.14 wt% of Ti_3_C_2_T_x_ within PVA fibers and it has great flexibility and mechanical strength. This reveals the great potential of the Ti_3_C_2_T_x_/CNC/PVA electrospun nanofiber composites for usage as flexible and wearable energy storage devices [30].

Polarity in PVA arises from the electronegativity difference among its atoms, for example, oxygen vs. carbon atoms. Conducting and nonconducting nanoparticles are typically used to obtain nanocomposites with high values of dielectric constant. Examples conducting fillers include graphene, carbon black, and carbon nanotubes; while the nonconducting ones include barium titanate, strontium titanate, and calcium titanate [43–46]. At certain concentration, percolation threshold, filler nanoparticles start to generate an interconnected network leads to an abrupt increase in the conduction. Percolation occurs at low concentrations of conducting fillers, like the Ti_3_C_2_T_x_ used herein, in comparison to non-conducting fillers. Fillers shape (zero-, one-, and two-dimensional), size, and orientation are among the parameters that determine the percolation threshold [43–46]. Ti_3_C_2_T_x_ as a two-dimensional material can easily achieve the percolation limits comparing to lower-dimensional fillers. In addition, orientation of the Ti_3_C_2_T_x_ nanosheets due to electrospinning could facilitate easier connection to form a continuum at lower concentration. This would explain the high conductivity obtained for sample C_0_M_2_, Fig 5, which contains only 0.14 wt% of Ti_3_C_2_T_x_.

The temperature dependence of the dc conductivity (σ_dc_) is presented in Fig 6. Values of σ_dc_ were calculated from the plateau values of the dc conductivity at low frequency, S6 Fig. DC conductivity accounts for the long-range hopping of the charge carriers and it involves a finite and reversible storage of charge in the material or at the interface [47].

**Fig 7:**
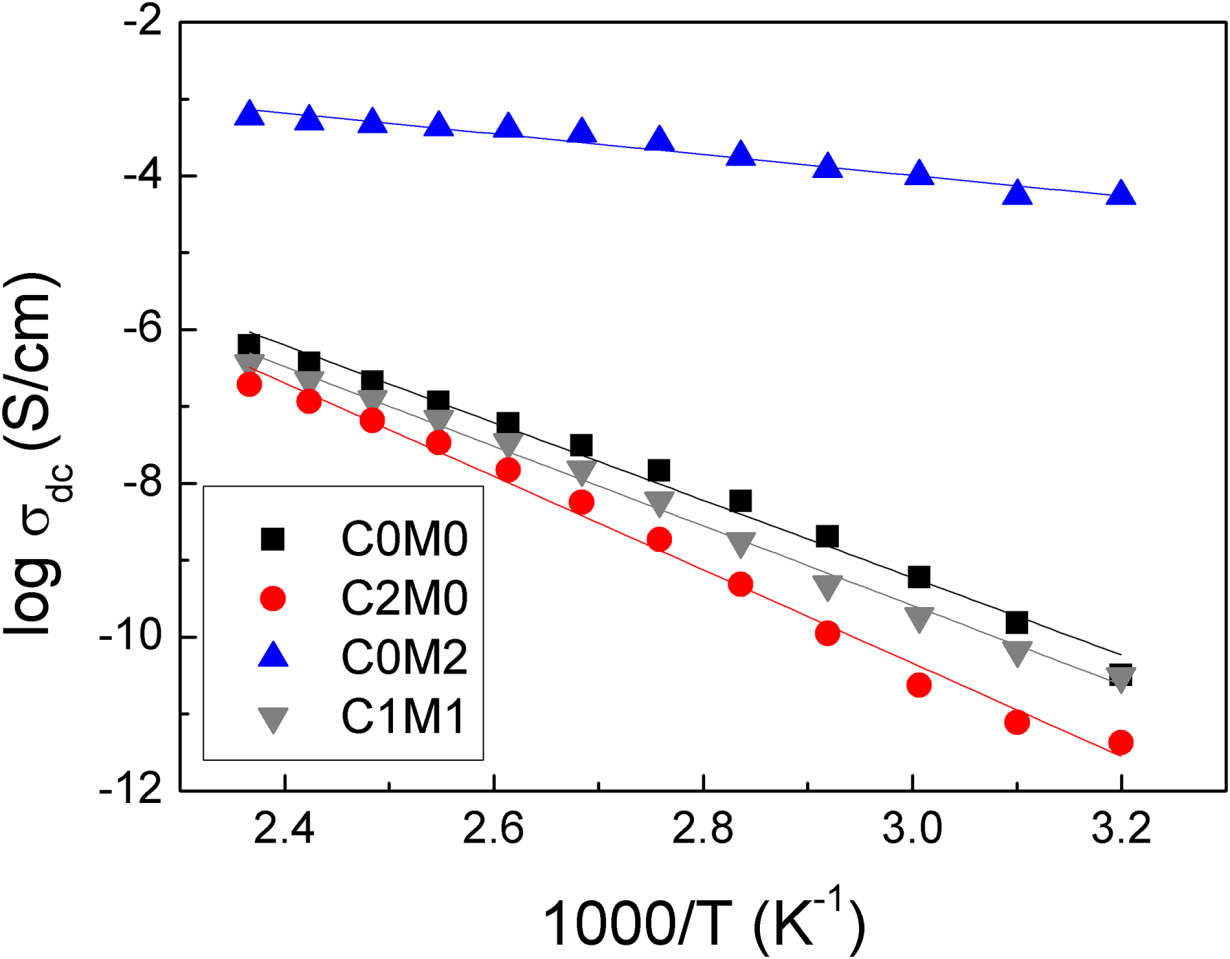
Temperature dependence of σ_dc_ for the Ti_3_C_2_T_x_/CNC/PVA nanofiber samples with different composition.

At low *f*, time scale or half period of oscillation (2*f*)^-1^, increases, charge carriers and ionic moieties can execute more elementary hops before the applied field reverses [48]. The monotonic upright raise of the plateau values, S6 Fig, with increasing temperature accounts for the dc conductivity increase especially above the polymer T_g_. The chain segmental mobility increases in the rubbery state and the charge carriers become more mobile as their motion is coupled to that of the chain segment. The temperature dependence of σ_dc_ in Fig 7 demonstrates strong Arrhenius behaviour according to the Eq 1:

$$\sigma_{dc}\;=\;\sigma_{o}\text{exp }\left(-\frac{E_{a}}{k_{B}T}\right)$$ (1)

Where σ_o_ is the pre-exponent factor and *k*_B_ is Boltzmann constant. The conductivity activation energy (*E*_a_) values extracted from Fig 7 are presented in Table 2.

**Table 2:** Conduction activation energy for the nanofiber composites.

Sample	Activation energy (eV)
C_0_M_0_	0.43
C_2_M_0_	0.52
C_0_M_2_	0.12
C_1_M_1_	0.45

The activation energy decreased as the content of the Ti_3_C_2_T_x_ in the composites was increased. It is interesting to note that for a PVA sample containing 14 wt% Ti_3_C_2_T_X_, the electronic conduction only required 0.02 eV of energy to launch however we were not able to obtain uniform fibers (data not shown). This low energy barrier reflects metallic or narrow band-gap semiconducting characteristics. Therefore, the electrospun composites presented herein could be a good candidate for electrode materials in Li-ion batteries [49]. In their theoretical study on Ti_3_C_2_ monolayer, Tang et al. predicted low diffusion barrier (0.07 eV) for an isolated Li atom on the Ti_3_C_2_ when compared to that for anatase TiO_2_ (0.35–0.65 eV) or graphite (~0.3 eV) [49]. This means that Li can exhibit faster transport and higher charge/discharge rates through the Ti_3_C_2_ monolayer comparing to TiO_2_ and graphite anodes. Our results for 14 wt% Ti_3_C_2_T_X_ electrospun composite confirm the theoretical prediction of low conduction barrier for this type of materials. However, Tang et al. also reported that surface functionalization of the Ti_3_C_2_ nanosheets by F or OH groups degrades the Li diffusion and decreases their Li storage capacity [50]. Our samples showed low conduction barrier although the Ti_3_C_2_ surface is functionalized with F or OH groups. Therefore, the conductivity enhancement obtained for our samples could be related to the electrospinning process used during their preparation. MXene based anode material in Li-ion batteries was reported to exhibit good capacity retention during 1000 galvanostatic charge/discharge cycles at rates up to 10°C [51].

## Conclusions

In conclusion, this work reports the preparation of Ti_3_C_2_T_x_, MXene and CNC reinforced electrospun PVA nanofibers for the first time. Diameter of obtained nanofibers ranged from 174 to 195 nm and varied depending on the ratio between PVA, Ti_3_C_2_T_x_ and CNC as measured by SEM and AFM microscopy. DMA showed an increase in the elastic modulus from 392 MPa for neat PVA fibres up to 855 MPa for fibres contain both fillers at temperature 25°C, which indicates a synergic reinforcing effect of both fillers. More particularly the electrical conductivity of the nanofiber composites increased with increasing content of Ti_3_C_2_T_x_, particularly for C_0_M_2_ sample (0.14 wt.% Ti_3_C_2_T_x_) where conductivity 0.8 mS×cm^-1^ was measured and showed narrow band-gap semiconducting characteristics, which makes them good candidates for electrode materials in Li-ion batteries. This could open window the development of a new class of conducting and mechanically enhanced nanofibers for energy applications.

## Supporting information

S1 Fig TEM images of fillers. A) CNC and B) Ti_3_C_2_T_x_.
(TIF)

S2 Fig XRD spectra of fillers. A) CNC and B) Ti_3_C_2_T_x_.
(TIF)

S3 Fig EDS spectra of electrospun mats. A) C_0_M_2_ and B) C_1_M_1_ sample.
(TIF)

S4 Fig PVA yarn. (TIF)

S5 Fig Sample positioning in tensile clamps (arrow indicate electrospun yarn). (TIF)

S6 Fig DC conductivity vs. frequency at different temperatures for electrospun mats. (a) C_0_M_0_ control and (b) C_0_M_2_ sample.
(TIF)

S1 File Supplementary section. (DOCX)
