# Supplementary figures and images for: 2D Ti3C2Tx (MXene)-reinforced polyvinyl alcohol (PVA) nanofibers with enhanced mechanical and electrical properties
Source: PLoS One. 2017 Aug 30;12(8):e0183705. doi: 10.1371/journal.pone.0183705 (PMC5576691; doi:10.1371/journal.pone.0183705)

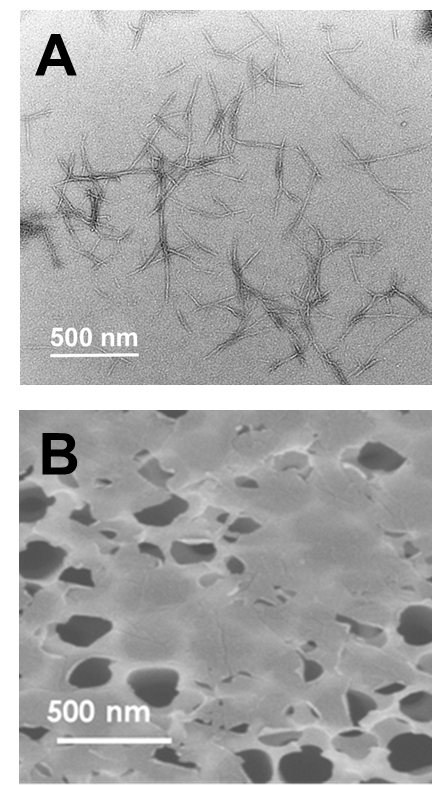

Supplement: S1 Fig — A) CNC and B) Ti3C2Tx. (TIF) [file pone.0183705.s001.tif]

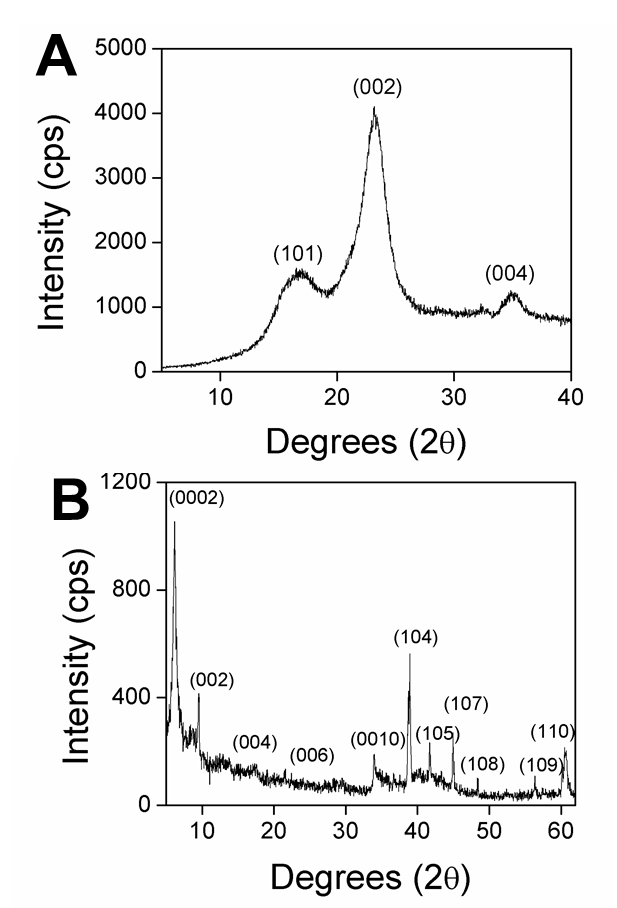

Supplement: S2 Fig — A) CNC and B) Ti3C2Tx. (TIF) [file pone.0183705.s002.tif]

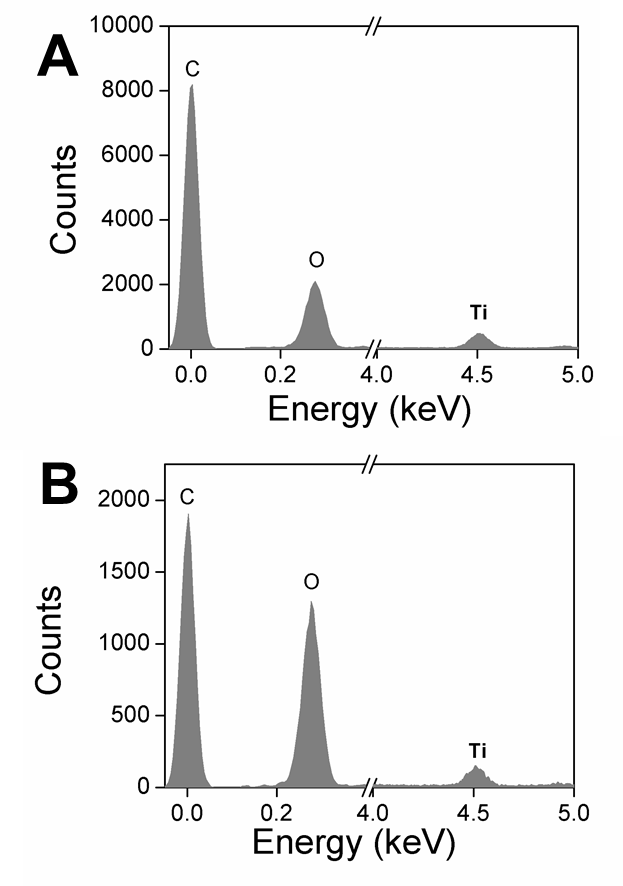

Supplement: S3 Fig — A) C0M2 and B) C1M1 sample. (TIF) [file pone.0183705.s003.tif]

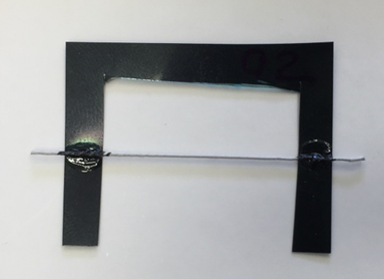

Supplement: S4 Fig — (TIF) [file pone.0183705.s004.tif]

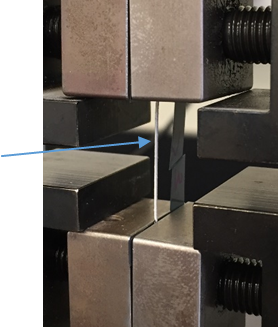

Supplement: S5 Fig — (TIF) [file pone.0183705.s005.tif]

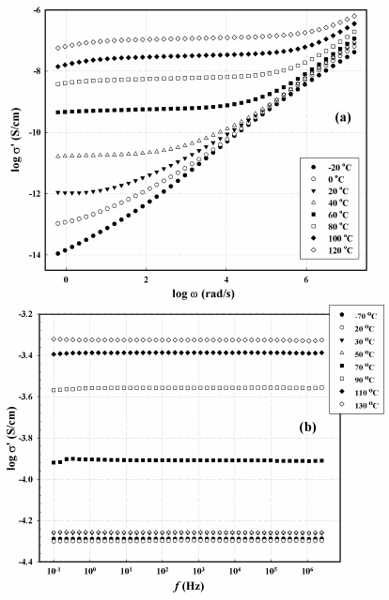

Supplement: S6 Fig — (a) C0M0 control and (b) C0M2 sample. (TIF) [file pone.0183705.s006.tif]
