# Supplementary material for: 2D Ti3C2Tx (MXene)-reinforced polyvinyl alcohol (PVA) nanofibers with enhanced mechanical and electrical properties
Source: PLoS One. 2017 Aug 30;12(8):e0183705. doi: 10.1371/journal.pone.0183705 (PMC5576691; doi:10.1371/journal.pone.0183705)
Supplement: S1 File — (DOCX) [file pone.0183705.s007.docx]

# Supplementary Section

Patrik Sobolčiak^1^, Adnan Ali^2^, Mohammad K. Hassan^1^, Mohamed I. Helal^2^, Aisha Tanvir^1^, Anton Popelka^1^, Mariam A. Al-Maadeed^1,3^, Igor Krupa^4^, Khaled A. Mahmoud^2,5^*

*^1^Center for Advanced Materials, Qatar University, P. O. BOX 2713, Doha, Qatar.*

*^2^Qatar Environment and Energy Research Institute (QEERI), Hamad Bin Khalifa University (HBKU), P. O. Box 5825, Doha, Qatar.*

*^3^Materials Science and Technology Program, Qatar University, P. O. BOX 2713, Doha, Qatar.*

*^4^QAPCO Polymer Chair, Center for Advanced Materials, Qatar University, P.O. Box 2713 Doha, Qatar.*

*^5^Department of Physics & Mathematical Engineering, Faculty of Engineering, Port Said University, 42523 Port Said, Egypt*

* Corresponding author Email: [kmahmoud@hbku.edu.qa](mailto:kmahmoud@hbku.edu.qa), Fax: (+974) 4454-41528

## Fillers characterization

The morphology and size of the CNC were analyzed by TEM (JEOL JEM-2100F (Japan)), operated at a voltage of 80 kV. Drops of the 0.01 wt.% CNC water suspension were deposited on carbon grids and dried before the analysis.

S1a Fig contains TEM images of CNC obtained from date palm leaves. CNC image proved successful destruction cellulose microfibers together with removal of lignin hemicellulose and another cementing material of cellulose fibers [1]. Average thickness of the CNC were estimated to be 14 nm (±5) and length 354 (±55) with high aspect ratio (over 20). S1b Fig showed morphology of Ti_3_C_2_T_x_ particles.

***Insert figure***

**S1 Fig. TEM images of fillers**

A) CNC and B) Ti_3_C_2_T_x_

XRD patterns were recorded with a powder diffractometer (Rigaku SmartLab) using Cu Ka radiation (λ=1.54 A˚) with 0.03^o^ 2θ steps.

XRD patterns of sample produced by etching in LiF/HCl solution are shown in S2b Fig The pattern is for multilayer Ti_3_C_2_T_x_, showing a sharp, intense peak (0002) at 6.17^o,^ which is at much lower angle that that typical of Ti_3_C_2_T_x_ produced by HF. Peaks for Ti_3_AlC_2_ (104), (105), (107), (108), (109) and (110) are still present, which proves that it’s a mix phase. It can be noticed that peaks for Ti_3_AlC_2_ traces have been shifted to lower angle which is due to increase in d-spacing between the planes.

***Insert figure***

**S2 Fig. XRD spectra of fillers**

A) CNC and B) Ti_3_C_2_T_x_

***Insert figure***

**S3 Fig. EDS spectra of electrospun mats**

A) C_0_M_2_ and B) C_1_M_1_ sample

S3 Fig illustrates the EDS spectra tracking of C, O and Ti within C_0_M_2_ and C_1_M_1_ nanofibers. EDS analysis confirmed the incorporation of Ti_3_C_2_T_X_ even at a low concentration of Ti_3_C_2_T_X_ within a blends.

## Tensile measurement

Tensile deformation was applied to various PVA electrospun mats in order to obtain information about mechanical performance of the composites. Due to the fact that the samples were composed of fibers and overall thickness of the samples did not exceed 100 µm, twisting of 5 cm wide and 20 cm long narrow strip of electrospun mats were performed [2]. Samples were gently stretched and twisted for 20 seconds to form nanofiber yarns, then attached to C shape holder using glue (S4 Fig).

***Insert figure***

**S4 Fig. PVA yarn**

C shape holder with twisted samples in the middle were placed between clamps and subsequently long side of C shape holder were cut by scissors prior to the measurement. S5 Fig illustrates sample positioning in tensile clamps.

***Insert figure***

**S5 Fig. Sample positioning in tensile clamps (arrow indicate electrospun yarn)**

## Nanofibers composites conductivity characterization

The dc conductivity of the control C_0_M_0_ and the C_0_M_2_ composite samples at different temperatures is depicted in S6 Fig.

***Insert figure***

**S6 Fig. DC conductivity vs. frequency at different temperatures for electrospun mats**

(a) C_0_M_0_ control and (b) C_0_M_2_ sample

**References**

[] Brinchi L, Cotana F, Fortunati E, Kenny JM. Production of nanocrystalline cellulose from lignocellulosic biomass: technology and applications. Carbohydr. Polym., 2013;94:154–169.

[2] Albertson AE, Teule F, Weber W, Yarger JL, Lewis RV, Effects of different post-spin stretching conditions on the mechanical properties of synthetic spider silk fibers. J. Mech. Behav. Biomed. Mater. 2014;29:225–234.
